# Upregulation of death receptor 5 and activation of caspase 8/3 play a critical role in ergosterol peroxide induced apoptosis in DU 145 prostate cancer cells

**DOI:** 10.1186/s12935-014-0117-5

**Published:** 2014-11-30

**Authors:** Jonghyun Han, Eun Jung Sohn, Bonglee Kim, Sunhee Kim, Gunho Won, Sangwook Yoon, Jihyun Lee, Moon Joon Kim, Hojin Lee, Kyujin Chung, Sung-hoon Kim

**Affiliations:** Cancer Preventive Material Development Research Center, College of Oriental Medicine, Kyung Hee University, Hoegidong, Dongdaemungu, Seoul, 130-701 Republic of Korea

**Keywords:** Ergosterol peroxide, Apoptosis, Caspase 8/3, Z-IETD-FMK, DR 5, DU 145 prostate cancer cells

## Abstract

**Background:**

Though ergosterol peroxide (EP) derived from Neungyi mushrooms (*Sarcodon aspratus*) was known to have cytotoxic, apoptotic, anti-inflammatory and antimycobacterial effects, the underlying molecular mechanism of EP still remains unclear. Thus, in the present study, the apoptotic mechanism of EP was elucidated in DU 145 prostate cancer cells.

**Methods:**

Cell viability of prostate cancer cells was measured by MTT assay. To see whether EP induces the apoptosis, FACS, western blot and TUNEL assay were performed. To determine the role of Death receptor (DR) 5 molecules in EP-induced apoptosis in DU 145 prostate cancer cells, the silencing of DR 5 was performed by using siRNAs.

**Results:**

EP showed significant cytotoxicity against DU 145, PC 3, M2182 prostate cancer cells. Also, EP effectively increased the sub G1 population and terminal deoxynucleotidyl transferase DUTP nick end labeling (TUNEL) positive cells in DU 145 prostate cancer cells. Furthermore, western blotting revealed that EP cleaved poly (ADP-ribose) polymerase (PARP) and caspase 8/3, attenuated the expression of fluorescence loss in photobleaching (FLIP), Bcl-_X_L and Bcl-2 as well as activated Bax, Fas-associated death domain (FADD) and DR 5 in a concentration dependent manner in DU 145 prostate cancer cells. Conversely, caspase 8 inhibitor Z-IETD-FMK blocked the apoptotic ability of EP to cleave PARP and an increase of sub G1 population in DU 145 prostate cancer cells. Likewise, the silencing of DR 5 suppressed the cleavages of PARP induced by EP in DU 145 prostate cancer cells.

**Conclusion:**

Overall, our findings suggest that ergosterol peroxide induces apoptosis via activation of death receptor 5 and caspase 8/3 in DU 145 prostate cancer cells as a cancer chemopreventive agent or dietary factor.

## Introduction

Prostate cancer is the second most frequently diagnosed cancer and the sixth leading cause of cancer death in males worldwide in 2011 [1]. Human prostate cancer cell lines are established including androgen dependent cancer LNCap and androgen independent cancers such as PC 3, TSU-Prl and DU 145 prostate cancer cells [2]. Though modern therapies such as chemotherapy, radiotherapy, surgery and castration have contributed to the treatment and prevention of prostate cancers, prostate cancer still remains refractory disease. Thus, recently herbal medicine [3,4] and phytochemicals [5–8] are attractive as supplements for combination therapy with cancer preventive or therapeutic agents targeting apoptosis, angiogenesis and metastasis.

Apoptosis is the process of programmed cell death (PCD), generally consisting of intrinsic mitochondrial pathway and extrinsic cell death pathway. Thus, recently anticancer agents from natural products are attractive by targeting apoptosis in several cancers [9–11]. Sarcodon edible mushroom [12] and its compound ergosterol peroxide (EP) [13], one of β-D-glucan metabolites, were reported to have antitumor activity in several cancers. Nonetheless, the underlying molecular mechanism of EP was not fully understood in prostate cancer. Thus, in the present study, the underlying apoptotic mechanism of EP was investigated in DU 145 prostate cancer cells targeting extrinsic apoptosis via DR 5 signaling using MTT assay, cell cycle analysis, TUNEL assay, western blotting and inhibitor study using caspase 8 inhibitor Z-IETD-FMK and siRNA transfection of DR 5.

## Methods

### Isolation of ergosterol peroxide

**Figure 1:**
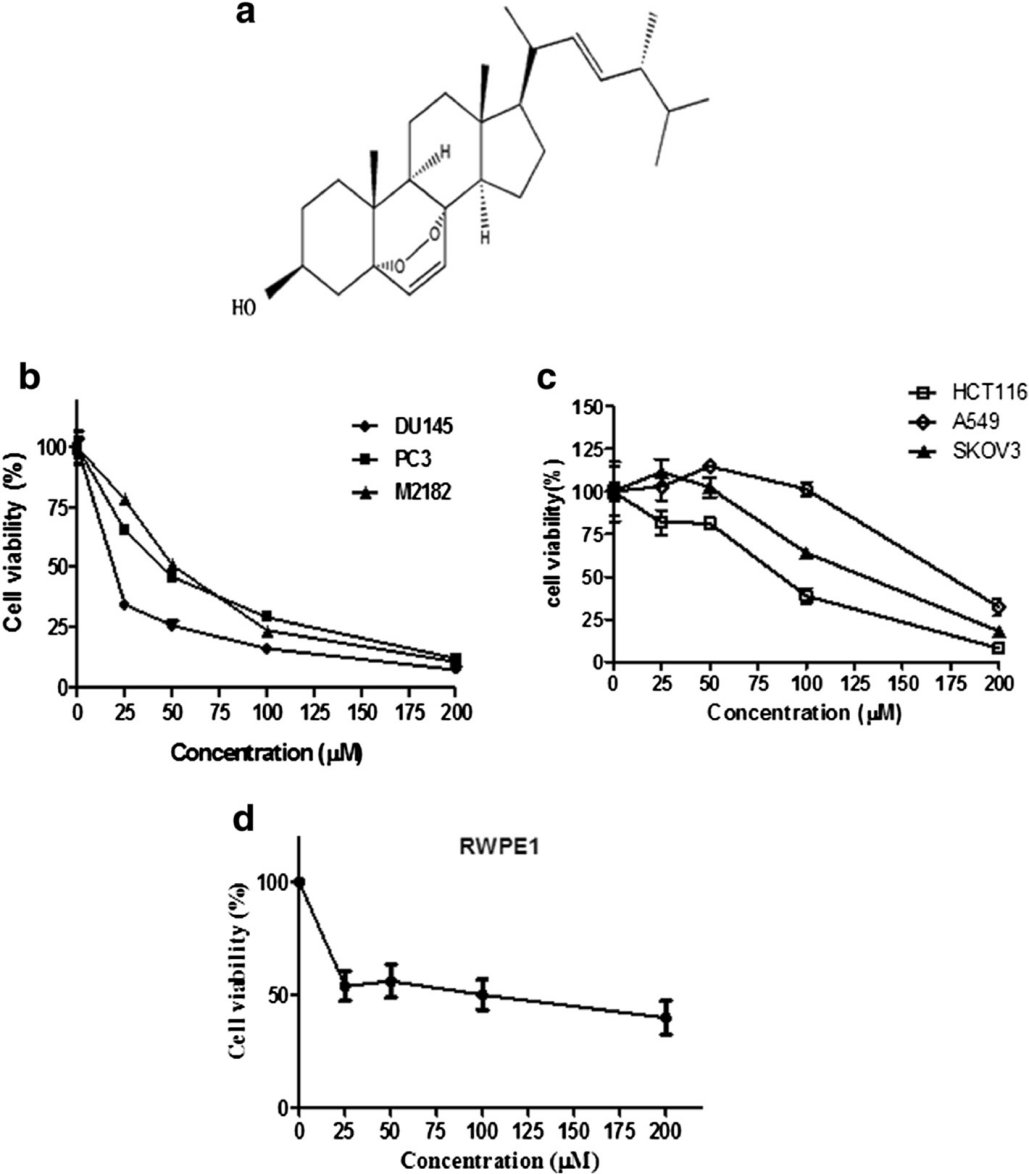
**Cytotoxic effect of EP against DU 145 prostate cancer cells.** The cells were treated with various concentrations of EP (0, 25, 50, 100, and 200 μM/mL) for 72 h. Then MTT assay was carried out. **(a)** Chemical structure of EP. **(b)** Cytotoxicity of EP in DU 145, PC 3 or M2182 prostate cancer cells and **(c)** HCT116 colon cancer cells, A549 lung cancer cells, SKOV3 ovary cancer cells, and **(d)** RWPE1 (prostate epithelial cells). Data are presented as means ± S.D of triplicate samples.

Ergosterol peroxide (EP; Figure 1) was isolated from Neungyi mushrooms (*Sarcodon aspratus*) as previously described with some modifications [12,14]. In brief, *Sarcodon aspratus* was extracted with acetone, and then EP was isolated from the acetone extract by using column chromatography (Waters, USA), silica gel column chromatography (Merck, Germany) and reverse-phase HPLC using a C18 column. Ergosterol was precipitated from the eluate of the silica gel column and finally EP was identified as 5-alpha, 8-alpha-epidioxy-22eE-ergosta-6, 22-dien-3beta-ol with chemical structure shown in Figure 1 by UV spectroscopy, mass spectrometry and ^13^C- and ^1^H-NMR. The purity of EP used in this experiment was over 97%.

#### Cell culture

DU 145, PC 3, M2182 prostate cancer cells, HCT116 colon cancer cells, A549 lung cancer cells, SKOV3 ovary cancer cells (ATCC® HTB-81™), and RWPE1 normal benign prostate epithelial cell lines were purchased from American Type Culture Collection (ATCC, Manassas, VA, USA). These cells were cultured in RPMI 1640 medium (Invitrogen, Carlsbad, CA, USA) supplemented with 10% FBS, 2 mML glutamine, and 100 units/ml antibiotic-antimycotics.

#### Cytotoxicity assay

The cell viability was evaluated by using MTT (3-(4,5-dimethylthiazol-2-yl)-2,5-diphenyltetrazolium bromide) assay (Sigma, St. Louis, MO, USA). DU 145 prostate cancer were seeded at 1 × 10^4^ cells/well of 96-well flat bottom plate and treated with various concentrations of EP (0, 25, 50, 100, 150 and 200 μg/ml) for 72 h. MTT was added to each well and incubated for 4 h at 37°C. Formazen crystals were dissolved by addition of dimethyl sulfoxide (DMSO) solution. The absorbance of each well was determined using the microplate reader (Molecular Devices Co., Sunnyvale, CA, USA) at 570 nm.

#### Cell cycle analysis

DU 145 prostate cancer cells treated with EP (25 and 50 μM) for 24 h were fixed in 75% ethanol at -20°C, resuspended in PBS containing RNase A (1 mg/ml), and incubated for 1 h at 37°C. Fixed cells were stained with propidium iodide (50 μg/ml) for 30 min at room temperature in dark. The DNA contents of the stained cells were analyzed using CellQuest Software with the FACSCalibur flow cytometry (Becton Dickinson, Franklin Lakes, NJ, USA). For the inhibitor study, caspase 8 inhibitor Z-IETD-FMK (20 μM) was treated in DU 145 prostate cancer cells in the presence or absence of EP for 24 h for cell cycle analysis.

#### TUNEL assay

Individual apoptotic cell death was observed using Dead End TM fluorometric terminal deoxynucleotidyl transferase DUTP nick end labeling (TUNEL) assay kit (Sigma, St. Louis, MO, USA) according to the manufacturer’s instructions. Briefly, DU 145 prostate cancer cells treated with EP (25 and 50 μM) were washed with cold PBS. Cells were seeded after fixing with 4% paraformaldehyde for 30 min and washed twice with PBS for 2 min. Resuspended cells in permeabilization solution (0.1% Triton X-100 and 0.1% Sodium citrate) for 4°C overnight were washed with PBS twice. The cells in 25 ml of TUNEL assay mixture were incubated for 60 min at 37°C in a humidified atmosphere in the dark. The TUNEL-stained cells were counter-stained with propidium iodide (Sigma, St. Louis, MO, USA) and visualized at x200 magnification by fluorescence microscopy (AXIO observer A1, Zeiss, Weimar, Germany).

#### Western blotting

Whole cell lysates of DU 145 were prepared by using lysis buffer (50 mM Tris-HCl, pH 7.4, 300 mM NaCl, 0.5% Triton X-100, 5 mM EDTA, 1 mM Na_3_VO_4_, 1 mM NaF, 10 μg/ml aprotinin, 10 μg/ml leupeptin, 10 μg/ml pepstatin, 10 mM iodoacetamide, 2 mM PMSF). To measure the protein contents, a Bio-Rad DC protein assay kit II (Bio-Rad, Hercules, CA, USA) was used. The proteins were separated on 10% tris-glycin gels, and electrotransferred onto a Hybond ECL transfer membrane with transfer buffer (25 mM Tris, 250 mM glycine, 20% methanol). The membranes were blocked in 5% nonfat dry milk in TBS buffer containing 0.1% Tween 20 (TBST) and immunoblotted with antibodies of cleaved caspase 3, cleaved caspase 8, procaspase 8, procaspase 9, PARP, Bcl-2, Bcl-_X_L, Bax, DR 5, FLIP and FADD (Cell signaling, Beverly, MA, USA), and β-actin (Sigma Aldrich Co., St. Louis, MO, USA), and then exposed to horseradish peroxidase (HRP)-conjugated secondary anti-mouse or rabbit antibodies (AbD Serotec, Raleigh, NC, USA). Protein expression was examined by using enhanced chemiluminescence (ECL) system (Amersham Pharmacia, Piscataway, NJ, USA).

#### siRNA DR 5 transfection

DU 145 prostate cancer cells were transiently transfected with a validated scrambled control siRNA, or siRNA specifically for DR 5 (Santa Cruz Biotechnology, Santa Cruz, CA, USA) by using Interferin™ transfection reagent (Polyplus-transfection Inc., New York, NY, USA). Briefly, the mixture of siRNA and Interferin™ transfection reagent was incubated for 10 min, added to each well of the cells (siRNA final concentration = 40 nM) and incubated at 37°C for 24 h before EP treatment.

### Statistical analyses

All data were expressed as means ± SD. The statistically significant differences compared with untreated group were calculated by Student’s *t*-test.

## Results

### Cytotoxic effect of EP against DU 145 prostate cancer cells

EP (Figure 1a) exerted significant cytotoxicity against DU 145 prostate cancer cells compared to non-treated cells. To evaluate cytotoxic effect of EP, MTT assay was performed. DU 145 prostate cancer were plated in 96-well plate and treated with various concentrations (0, 25, 50, 100, and 200 μM/ml) of EP for 72 h. As shown in Figure 1b, the cytotoxicity of EP was significantly exhibited in concentration dependent manner in DU 145, PC 3 and M2182 prostate cancer cells. Additionally, the cytotoxicity of EP was determined in A549 lung cancer cells, HCT116 colon cancer cells, SKOV3 ovary cancer cells (Figure 1c) and RWPE1 (normal prostate epithelial cells) (Figure 1d). MTT assay revealed that EP showed the strong cytotoxic effect in the prostate cancer cells lines such as DU 145, PC 3 or M2182 compared to A549 lung cancer cells, HCT116 colon cancer cells, SKOV3 ovary cancer cells or RWPE1 cells.

### Apoptotic effect of EP in DU 145 prostate cancer cells

**Figure 2:**
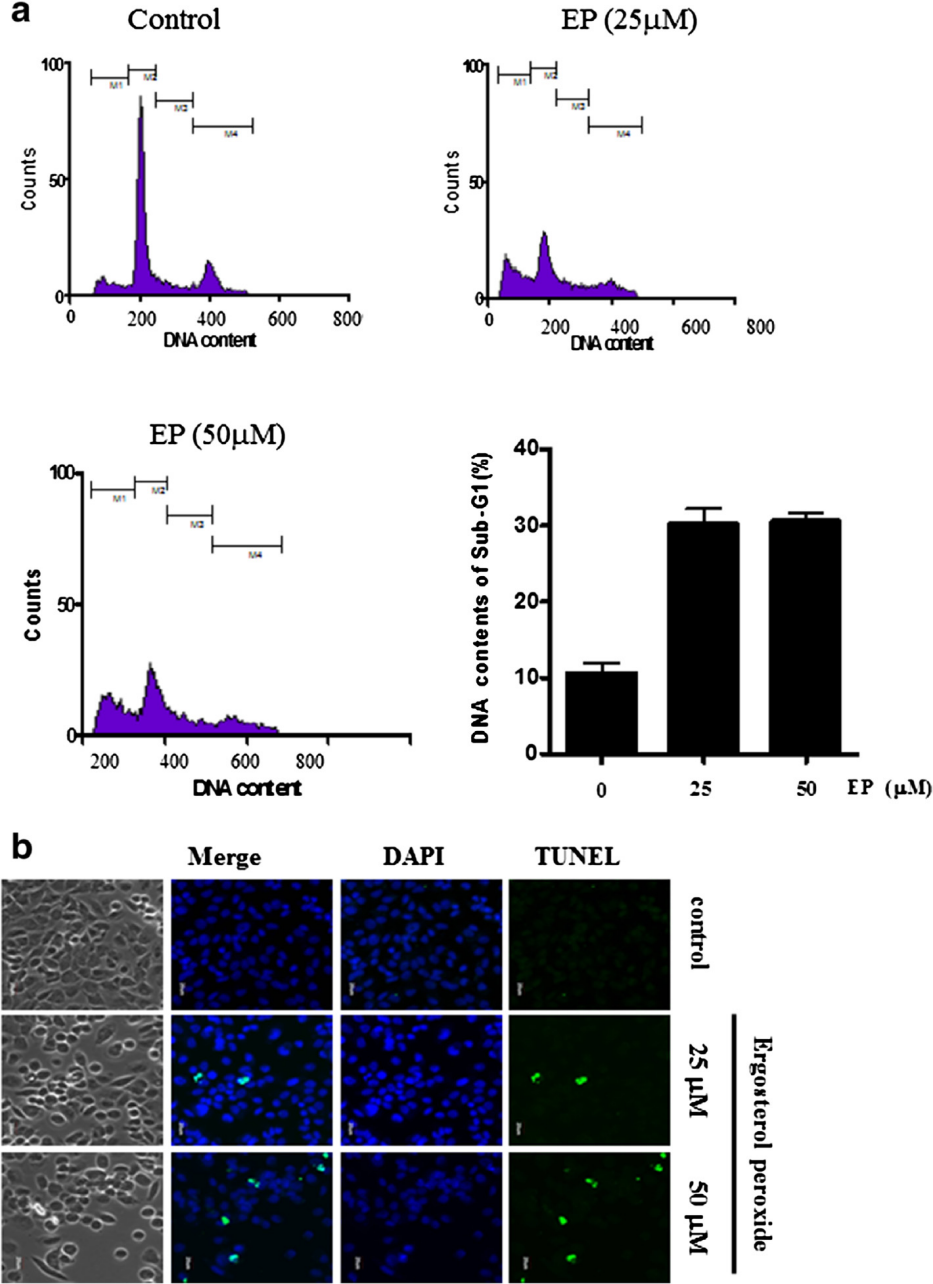
**The effect of EP inducing apoptosis in DU 145 cells by cell cycle and TUNEL assay. (a)** The effect of EP on sub G1 population in DU 145 cells. DU 145 prostate cancer were exposed to with EP (25 and 50 μM) for 24 h and stained with propidium iodide (50 μg/ml) for 30 min at room temperature in dark. Then flow cytometric analysis was performed. Data are presented as means ± S.D of triplicate samples. **(b)** Effect of EP on TUNEL assay in DU 145 cells. DU 145 cells treated with EP (25 and 50 μM) were seeded after fixing with 4% paraformaldehyde for 30 min and incubated in 25 ml of TUNEL assay mixture for 60 min at 37°C in the dark. TUNEL-stained cells were counter-stained with propidium iodide (Sigma, St. Louis, MO, USA) and visualized at x200 magnification by fluorescence microscopy.

To confirm whether the cytotoxicity of EP was due to apoptosis, cell cycle analysis and TUNEL assay were performed. In the present study, EP significantly increased sub G1 population (Figure 2a) and also TUNEL positive green colored cells as one of apoptosis features in DU 145 prostate cancer cells (Figure 2b).

### Effect of EP on apoptosis related proteins in DU 145 prostate cancer cells

**Figure 3:**
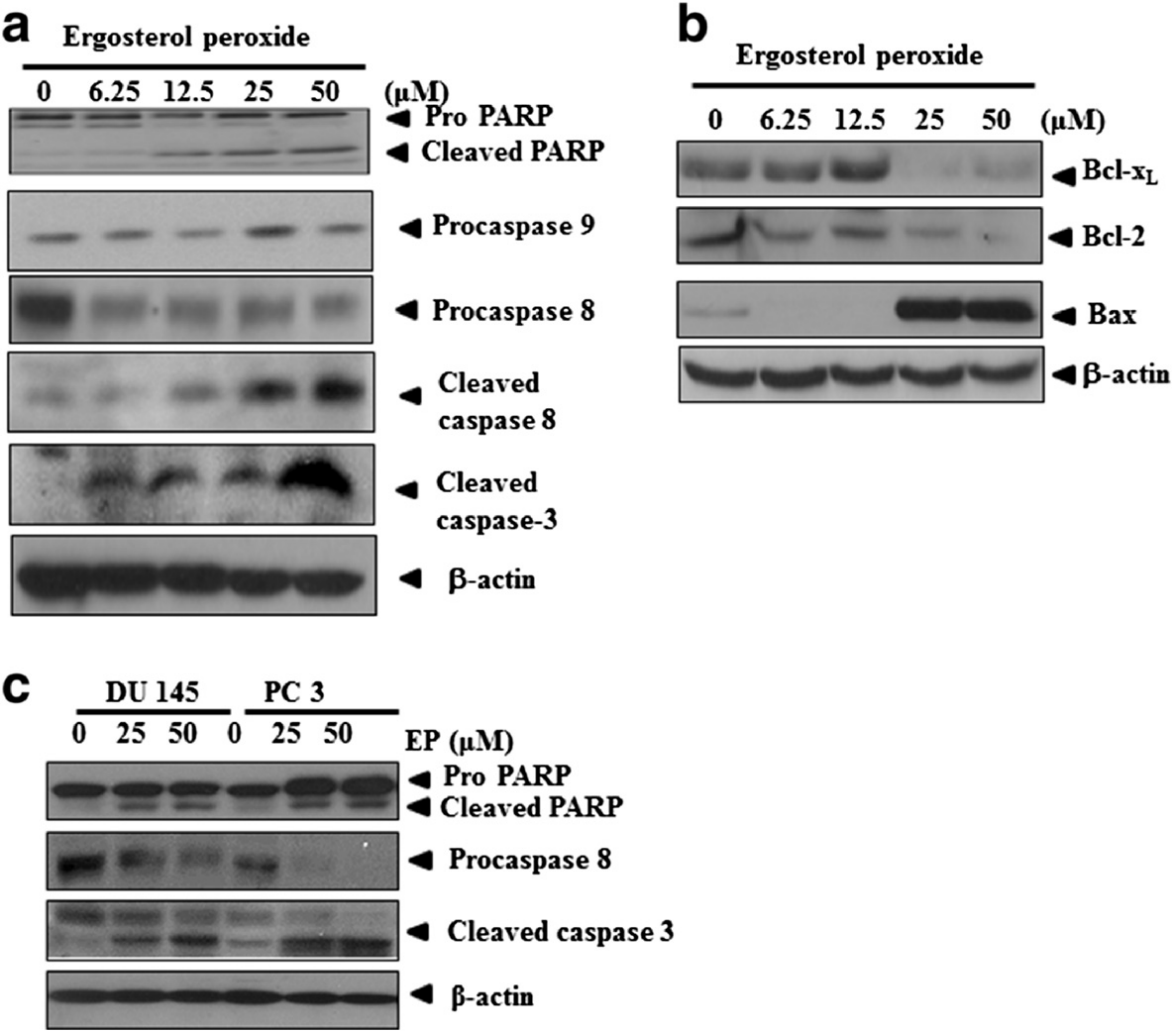
**Effect of EP on PARP, procaspase 8/9, cleaved caspase 8/3, Bcl-2, Bcl-**_**XL**_ **and Bax in DU 145 prostate cancer cells. (a, b)** DU 145 cells were treated with various concentrations of EP (0, 25, 50, 100, and 200 μM) for 72 h and western blot was carried out with Bcl-x_L_, Bcl-2, Bax, or β-actin antibodies to see apoptosis or survival related genes. **(c)** DU 145 and PC 3 cells were treated with EP (25, 50 μM) and western blot was carried out with PARP, procaspase 8, cleaved caspase 3 or β-actin antibodies.

EP activated extrinsic apoptosis and attenuated survival genes in DU 145 prostate cancer cells. EP activated cleaved PARP, Bax and caspase 8/3, and attenuated the expression of procaspase 8, Bcl-2, Bcl-_X_L without affecting procaspase 9 from the concentration of 25 μM compared to untreated control in DU 145 prostate cancer cells as shown Figure 3a, and b. We also confirmed that EP treatment attenuated procaspase 8 and activated cleaved PARP and cleaved caspase 3 in PC 3 cells (Figure 3c).

### Caspase 8 inhibitor Z-IETD-FMK and siRNA DR 5 blocked the apoptotic ability of EP to cleave PARP

**Figure 4:**
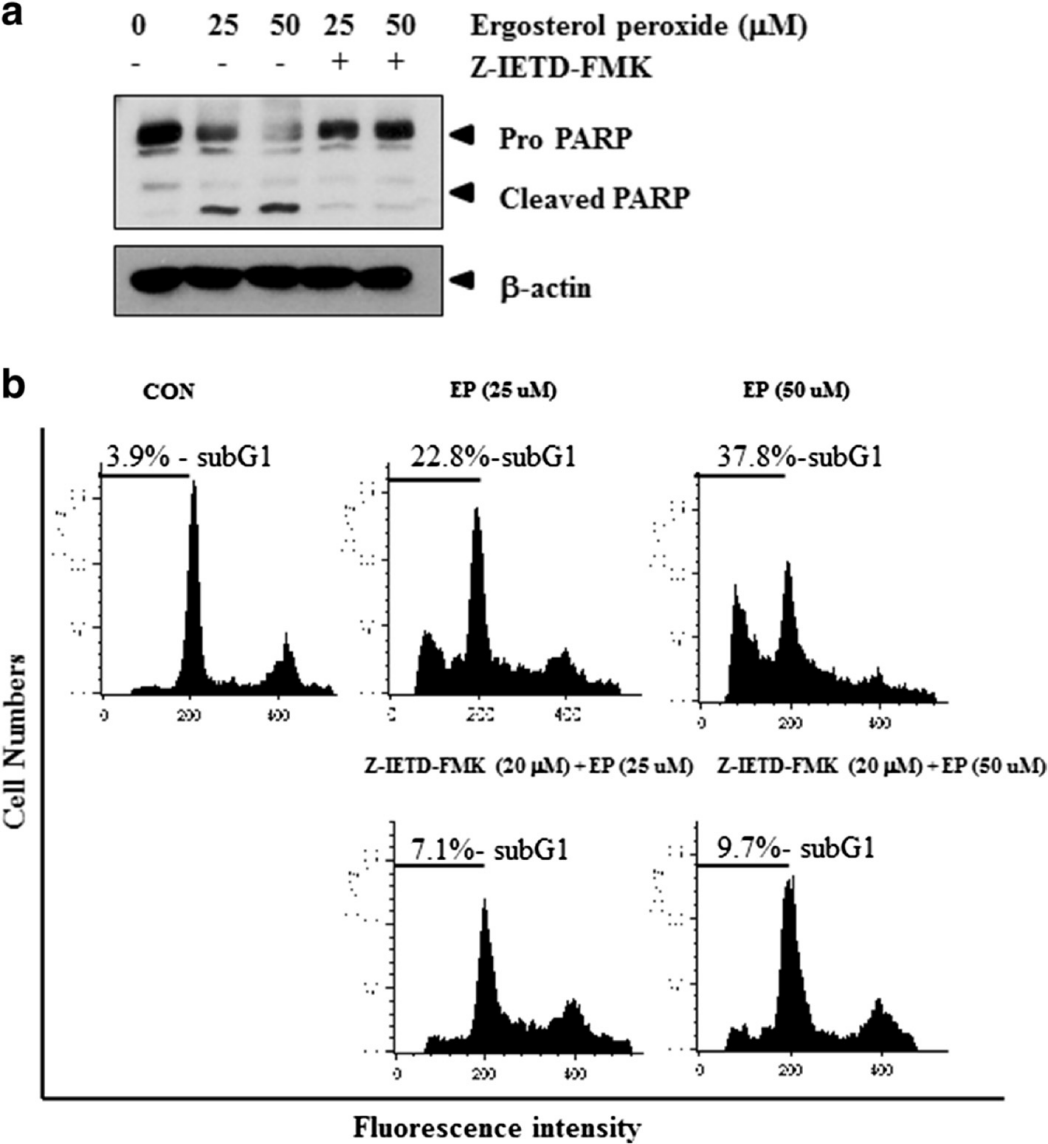
**Effect of caspase 8 inhibitor Z-IETD-FMK on PARP in EP treated DU 145 prostate cancer cells.** DU 145 cells were treated with EP (25 or 50 μM) and/or caspase 8 inhibitor Z-IETD-FMK. Western blot assay **(a)** and FACS analysis **(b)** were performed.

Caspase 8 inhibitor Z-IETD-FMK interrupted the cleavage of PARP induced by EP in DU 145 prostate cancer cells (Figure 4a). Consistently, Z-IETD-FMK suppressed sub G1 population up to 7.1% and 9.7%, respectively, at the concentrations of 25 and 50 μM in EP treated DU 145 prostate cancer cells, while EP enhanced sub G1 population up to 22.8% and 37.8% at the same concentrations in DU 145 prostate cancer cells (Figure 4b).

### Apoptotic ability of EP via caspase 8, DR 5 in DU 145 prostate cancer cells

**Figure 5:**
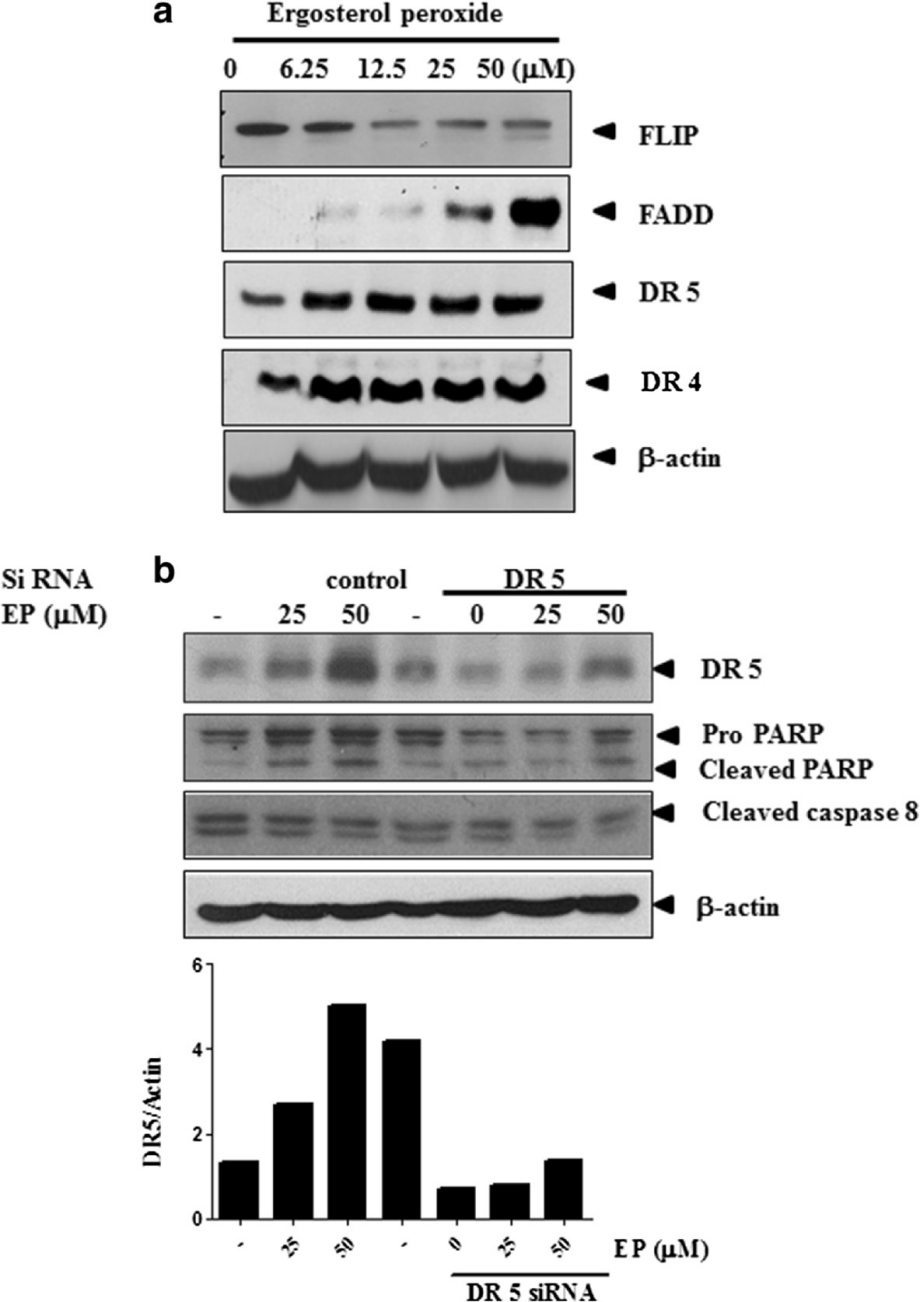
**Effect of siRNA DR 5 transfection on PARP and procaspase 8 in EP treated DU 145 prostate cancer cells. (a)** Western blot assay was carried out with indicated antibodies to see the expression of FLIP, FADD, DR 5 or DR 4 in EP treated DU 145 cells. **(b)** After transfection with siRNA DR 5, EP was treated and incubated in DU 145 cells for 24 h. The lysates were subjected to western blot analysis using DR 5, PARP, procaspase 8 or β-actin antibodies. Bar graph represents the relative expression of the DR 5 protein by using images J software.

EP treated DU 145 enhanced the expression of FADD, DR 5 and DR 4 while FLIP was attenuated (Figure 5a). To confirm the roles of caspase 8 and DR 5 molecules in EP induced apoptosis in DU 145 prostate cancer cells, the silencing of DR 5 by siRNAs were used. As shown in Figure 5b, siRNA transfection of DR 5 also blocked upregulation of DR 5 and cleavage of PARP and caspase 8 induced by EP in DU 145 prostate cancer cells (Figure 5b) indicating that DR 5 mediates EP induced apoptosis in DU 145 cells.

## Discussion

Neungyi mushrooms have been used as traditional medicine or healthy food for years. Recent studies revealed the multi-biological activities including immunomodulating, anti-inflammatory and anti-cancer effects of mushrooms such as *Phellinus linteus, Sarcodon asparatus, Ganoderma applanatum, Paecilomyces tenuipes* and *Agaricus blazei* [15–17].

Though ergosterol peroxide (EP) as a metabolite of β-glucan, a well known antitumor component from mushrooms was reported to have anti-inflammatory, anti-microbial, anti-tumor, anti-oxidative and immunosuppressive effects, the underlying antitumor mechanism of EP was not clearly understood in prostate cancer cells until now. Thus, to determine the potential of EP as a cancer chemopreventive agent or a dietary factor, in the current study, the apoptotic mechanism of EP was elucidated mainly in DU 145 prostate cancer cells.

EP showed the strong cytotoxic effect in the prostate cancer cells lines such as DU 145, PC 3 or M2182 compared to other cancer cells in a concentration dependent manner by MTT assay, implying its cytotoxic effect in prostate cancer cells. To confirm whether the cytotoxicity was induced through apoptosis induction, cell cycle analysis using flow cytometry and TUNEL assay was performed in DU 145 prostate cancer cells. EP significantly increased sub G1 population and the number of TUNEL positive green colored cells in DU 145 prostate cancer cells, indicating the apoptotic feature of EP since apoptosis accompanies with cell membrane wrinkled, DNA fragmentation, cytosol calcium increased and form the apoptotic body in the cells.

In general, there are two classical apoptotic pathways including intrinsic mitochondrial pathway and extrinsic death receptor pathway in cells [9]. In caspase-dependent mitochondrial pathway, cytochrome *c* activates Apaf-1 and procaspase 9, forming an apoptosome [18,19]. In contrast, the extrinsic signaling pathways initiate apoptosis via activation of caspase 8 and death receptors such as FasL/FasR, TNF-α/TNFR 1, Apo3L/DR 3, Apo2L/DR 4 and Apo2L/DR 5 [20], while death receptor-mediated apoptosis can be inhibited by a protein called c-FLIP which will bind to FADD and caspase 8 [21,22]. Western blotting showed that EP cleaved PARP and caspase 8/3 without affecting caspase 9, attenuated the expression of anti-apoptotic proteins such as c-FLIP, Bcl-_X_L and Bcl-2 as well as activated the expression of proapoptotic protein Bax, FADD and DR 5 in a concentration dependent manner in DU 145 prostate cancer cells, strongly demonstrating death receptor dependent apoptosis by EP in DU 145 prostate cancer cells.

Furthermore, to confirm EP induced extrinsic apoptosis, caspase 8 inhibitor Z-IETD-FMK and siRNA DR 5 transfection were used in inhibitor study for EP mediated apoptosis. Z-IETD-FMK blocked the apoptotic ability of EP to cleave PARP and an increase of sub G1 population and the silencing of DR 5 suppressed the cleavages of PARP induced by EP in DU 145 prostate cancer cells, indicating the death receptor dependent apoptosis induced by EP.

## Conclusions

In summary, EP showed significant cytotoxicity in DU 145 prostate cancer cells, increased sub G1 population and TUNEL positive cells, activated the cleavages of PARP and caspase 8/3 and Bax, FADD, and DR 5, but attenuated the expression of survival genes such as c-FLIP, Bcl-_X_L and Bcl-2 in DU 145 prostate cancer cells. Collectively, our findings suggest that activation of death receptor 5 and caspase 8/3 plays a key role in ergosterol peroxide induced apoptosis in DU 145 prostate cancer cells.
